# Evolutionary history expands the range of signaling interactions in hybrid multikinase networks

**DOI:** 10.1038/s41598-021-91260-w

**Published:** 2021-06-03

**Authors:** Philippe Ortet, Sylvain Fochesato, Anne-Florence Bitbol, David E. Whitworth, David Lalaouna, Catherine Santaella, Thierry Heulin, Wafa Achouak, Mohamed Barakat

**Affiliations:** 1 Aix Marseille Univ, CEA, CNRS, BIAM, LEMIRE, 13108 Saint Paul-Lez-Durance, France; 2 CNRS, Institut de Biologie Paris-Seine, Laboratoire Jean Perrin (UMR8237), Sorbonne Université, 75005 Paris, France; 3 Institute of Bioengineering, School of Life Sciences, Ecole Polytechnique Fédérale de Lausanne (EPFL), 1015 Lausanne, Switzerland; 4 Institute of Biological, Environmental and Rural Sciences, Aberystwyth University, Ceredigion, SY23 3DD UK; 5 CNRS, ARN UPR 9002, Université de Strasbourg, 67000 Strasbourg, France

**Keywords:** Microbiology, Bacteria, Bacterial evolution, Computational biology and bioinformatics, Gene regulatory networks

## Abstract

Two-component systems (TCSs) are ubiquitous signaling pathways, typically comprising a sensory histidine kinase (HK) and a response regulator, which communicate via intermolecular kinase-to-receiver domain phosphotransfer. Hybrid HKs constitute non-canonical TCS signaling pathways, with transmitter and receiver domains within a single protein communicating via intramolecular phosphotransfer. Here, we report how evolutionary relationships between hybrid HKs can be used as predictors of potential intermolecular and intramolecular interactions (‘phylogenetic promiscuity’). We used domain-swap genes chimeras to investigate the specificity of phosphotransfer within hybrid HKs of the GacS–GacA multikinase network of *Pseudomonas brassicacearum*. The receiver domain of GacS was replaced with those from nine donor hybrid HKs. Three chimeras with receivers from other hybrid HKs demonstrated correct functioning through complementation of a *gacS* mutant, which was dependent on strains having a functional *gacA*. Formation of functional chimeras was predictable on the basis of evolutionary heritage, and raises the possibility that HKs sharing a common ancestor with GacS might remain components of the contemporary GacS network. The results also demonstrate that understanding the evolutionary heritage of signaling domains in sophisticated networks allows their rational rewiring by simple domain transplantation, with implications for the creation of designer networks and inference of functional interactions.

## Introduction

Two-component signal transduction systems (TCSs) are mainly found in prokaryotes, where they control diverse and important adaptive responses to changes in environmental conditions^1^. The prototypical TCS comprises a pair of signal transduction proteins—a histidine kinase (HK) and cognate response regulator (RR)—both of which are multi-domain proteins. HKs contain sensor domains (input), which upon stimulation activate autophosphorylation of a histidine residue in the HK transmitter domain. The phosphorylated transmitter domain then binds to the receiver domain of the RR partner, leading to transfer of the phosphoryl group to an aspartate residue of the receiver domain. In the majority of cases, the RR phosphorylation changes the activity of an effector domain (output) within the RR, which brings about a physiological change, often through altered expression of specific genes. A subset of TCSs are non-prototypical systems, which include a class of TCSs designated ‘hybrid’ HKs^2^. Hybrid HKs have both transmitter and receiver domains, but variants are commonly found which possess additional signaling domains, for instance the ‘unorthodox’ sub-class of hybrid HKs have an additional histidine-containing phosphotransfer (HPt) domain.

The TCS gene sets in an organism can expand by duplication, divergence and lateral transfer, resulting in some species possessing several hundreds of such signaling proteins. This raises the question of how specificity is maintained between cognate partners despite the structural similarity common to all TCS proteins. It has been shown that for prototypical TCSs, the interaction between cognate protein partners is highly specific, preventing unwanted cross-talk between TCSs^3–5^. However, domain-domain interactions within hybrid HKs have relaxed domain-domain specificity and affinity compared to the interprotein interactions of prototypical HKs and RRs. Relaxed specificity is tolerable in hybrid HKs because the tethering of transmitter and receiver domains within a single protein increases the effective concentration of each domain in the vicinity of the other^6–8^.

Although prototypical TCS protein partners encoded by adjacent genes are frequent, isolated HK and RR genes (orphans) are also abundant, as are complex gene loci encoding multiple TCS proteins^9^. This makes the identification of signaling partnerships non-trivial. The P2CS database (http://www.p2cs.org) provides paired HK and RR phylogenetic trees, which shed light on evolutionary heritage and can additionally provide clues about protein HK-RR partnerships^10^. Indeed, HKs and RRs that are known to interact, fall into corresponding subfamilies^10–12^. This phylogenetic relationship is mainly attributed to the coevolution of the interacting domains of TCS partners^4,13^ and the need to prevent unwanted cross-talk between non-cognate TCSs.

Nevertheless, because of their shared evolutionary heritage, non-cognate interactions are possible between phylogenetically-related TCS domains^14–17^. Sometimes, observed ‘non-cognate’ interactions turn out not to be unwanted cross-talk, but rather are phenotypically important ‘cross-communication’ between TCS, forming signaling networks^14,18,19^. Phylogenetic analysis indicated that there was no common ancestor for hybrid HKs, and that their origin and expansion were achieved by lateral recruitment of a receiver domain into an HK molecule and then duplication as one unit^20^.

The majority of hybrid HK network interactions described in the literature concern the GacS-GacA TCS in the model bacterial species *Pseudomonas aeruginosa,* an opportunistic human pathogen causing respiratory infections of hospitalized patients. GacS is an unorthodox HK, which phosphorylates the response regulator GacA. GacA and GacS are part of a multikinase network, in which the LadS and RetS hybrid HKs affect the activation of GacA by GacS^18,19^. The GacS-GacA TCS network and additional hybrid HKs (PA1611, PA1976 and PA2824) control the expression of small regulatory RNAs (sRNAs), which orchestrate several functions in gammaproteobacteria^21–23^. In *P. aeruginosa*, phosphorylated GacA specifically and exclusively activates the expression of the sRNA genes *rsmY* and *rsmZ*^24^, whereas in *Pseudomonas fluorescens* CHA0, phosphorylated GacA activates the expression of four sRNA genes, *rsmX*, *rsmY*, *rsmZ*, and *rgsA*^25,26^. The deletion of *rsmY* and *rsmZ* genes in *P. aeruginosa*^24^ and of *rsmX, rsmY*, and *rsmZ* in *P. fluorescens* CHA0^25^ result in phenotypes that are similar to those of *gacS* and *gacA* mutants.

*Pseudomonas brassicacearum* was isolated as the major root-associated bacterial species in the rhizosphere of *Arabidopsis thaliana* and *Brassica napus*^27^. *P. brassicacearum* has the ability to suppress plant pathogens by producing antifungal compounds, such as 2,4-diacetylphloroglucinol and cyanide. The two-component GacS-GacA system by activating the expression of three small regulatory RNAs (RsmX, RsmY, RsmZ), positively controls the expression of secondary metabolites (*e.g.* the antifungal compounds DAPG and cyanide), plant hormones (*e.g.* auxin), exoenzymes (*e.g.* lipase and protease)^28^, AHLs, the type VI secretion machinery, alginate synthesis, and biofilm-forming ability^23^. Mutations in the *gacS*-*gacA* system result in drastic pleiotropic changes in *P. brassicacearum*^23^. This bacterial species combines effictive plant colonization^27,29^ with other beneficial attributes for plant health and growth, including release of plant growth hormones (such as indole-3-acetic acid)^23^, modulation of ethylene by the enzyme 1-aminocyclopropane-1-carboxylate deaminase^30^, production of two different siderophores^31^ and protection against phytopathogens by producing at least two antifungal compounds DAPG and cyanide^23^. The genome of *P. brassicacearum* contains homologues of the *P. aeruginosa* hybrid HKs, making it a relevant model for deciphering the GacS-GacA network.

Here, we report how the evolutionary relationships between hybrid HKs can be used as predictors of potential intermolecular interactions. We used domain-swap genes chimeras of *P.* *brassicacaerum* GacS to assay interactions between signaling domains of hybrid HKs predicted by their evolutionary relationships. As well as showing the utility of the ‘phylogenetic promiscuity’ approach, we propose that additional hybrid HKs might be involved in the GacS cascade that controls phytobeneficial traits, and suggest likely new signaling interactions within the GacS multikinase network. This approach also allows us to suggest potential intermolecular hybrid HK interactions in other bacteria using phylogenetic analyses available through the P2CS database.

## Results

### Kinase and receiver domains of ‘GacS cluster’ hybrid HKs have co-evolved

**Figure 1:**
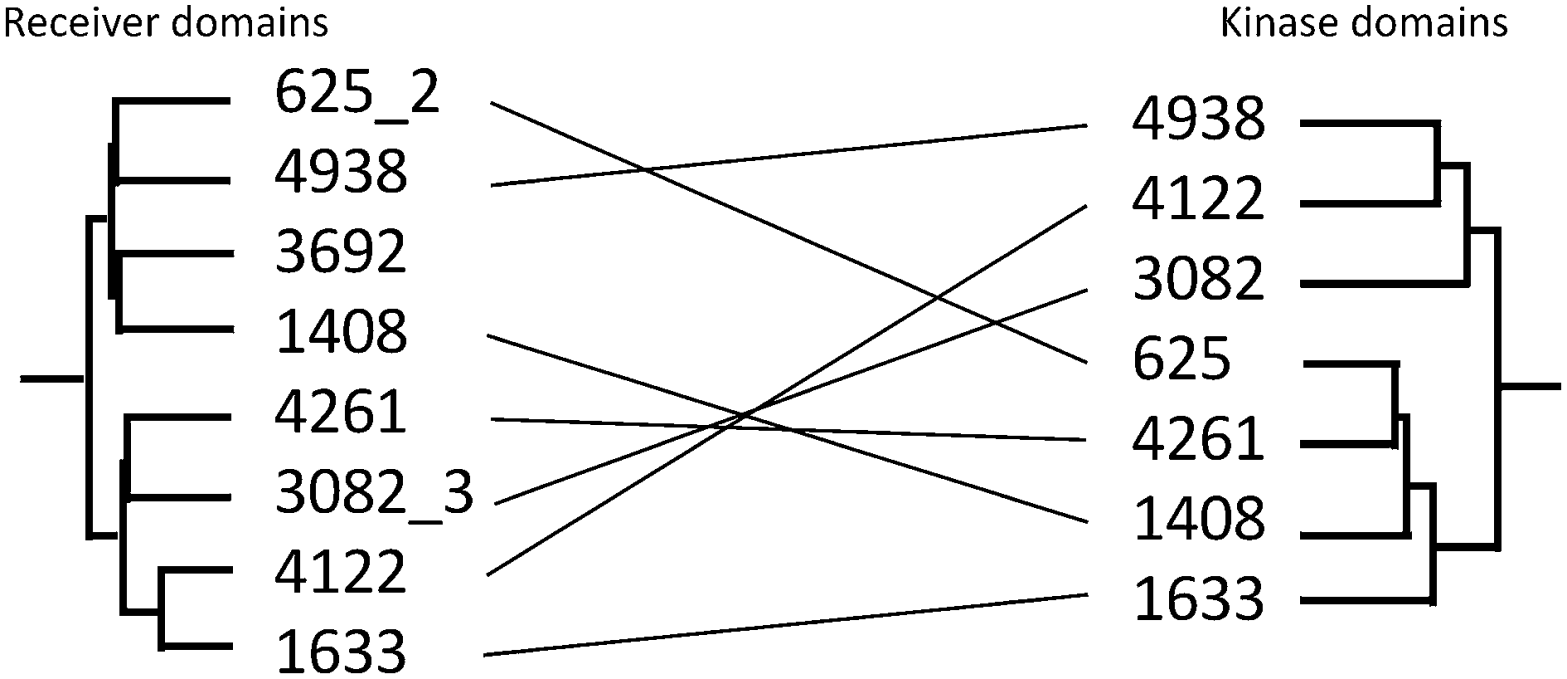
Paired phylogenetic trees from *Pseudomonas brassicacearum*, focusing on the ‘GacS cluster’. Black lines connect hybrid HKs to their internal receivers. A number is added to the protein identifier to indicate the position of the receiver, in the case of multiple receiver domains (i.e. 3082_3: third receiver of 3082). All the HKs of the GacS cluster are transmembrane proteins.

**Figure 2:**
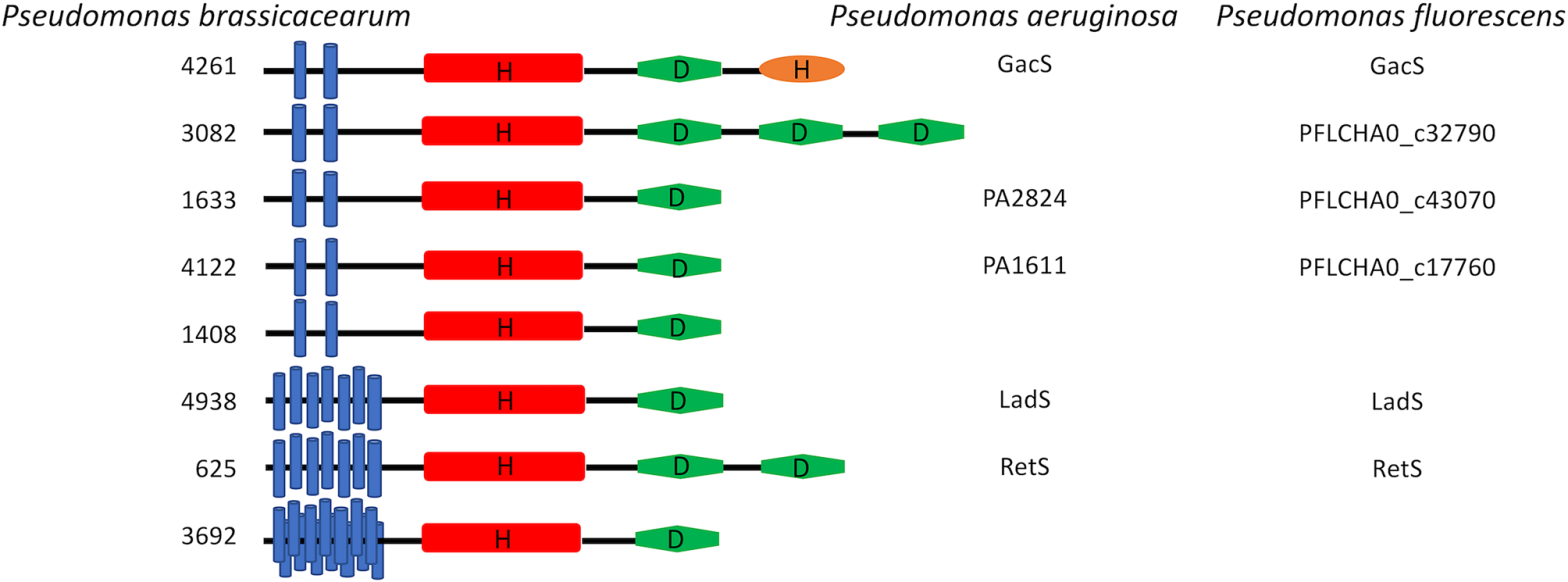
Schematic representation of the conserved domains of each hybrid sensor kinase belonging to the ‘GacS cluster’. Homologues of the *P. brassicacearum* hybrid HKs, found in the *P. aeruginosa* and *P. fluorescens* genomes, are specified. Kinase domain (red), receiver domain (green), HPt domain (orange), transmembrane segment (blue). Histidine (H) and aspartate (D) indicate phosphorylation sites.

In the latest version of the P2CS database, paired phylogenetic trees were implemented for evolutionary analysis and identification of candidate partners for orphan TCS proteins^10^. The genome of *P. brassicacearum* NFM421^30^ was added to P2CS and analyzed to produce trees of kinase and receiver domains from encoded TCS proteins (Supplementary Fig. S1). The kinase domain of *P. brassicacearum* Psebr_a4261 (GacS) was found in a cluster (the GacS kinase cluster) alongside seven other hybrid HKs: Psebr_a4938 (LadS), Psebr_a625 (RetS), Psebr_a1633, Psebr_a1408, Psebr_a3082 and Psebr_a4122 (PA1611) (Fig. 1, protein identifiers are abbreviated in the figure and in the rest of the manuscript). These seven proteins also cluster together in the *P. brassicacearum* receiver domain tree (the GacS receiver cluster), which also includes the receiver domain of 3692 (Fig. 1), another hybrid HK which is encoded adjacent to a further receiver domain-containing protein (the LuxR-family RR, 3693). Protein 3692 is also included, if we slightly extend domain kinase cluster of GacS (Supplementary Fig. S1). Two hybrid HKs include more than one receiver domain, RetS contains two receivers (625_1 and 625_2) and 3082 contains three. The receiver domains 625_1, 3082_1 and 3082_2 do not belong to the GacS cluster. Homologues of the *P. brassicacearum* hybrid HKs are found in the *P. aeruginosa* genome (4261 = GacS, 4938 = LadS, 625 = RetS, 1633 = PA2824 and 4122 = PA1611) (Fig. 2).

We further applied Direct Coupling Analysis (DCA), a statistical inference method based on coevolution^13,32,33^, to compute the interaction scores (specifically, effective interaction energies^34^) between the *P.* *brassicacearum* GacS HK domain and all the receiver domains involved in *P.* *brassicacearum* hybrid HKs. Small values of this interaction score have proved successful at predicting the correct interaction partners among prototypical cognate HK–RR pairs, through an iterative pairing algorithm (IPA) which starts from unpaired sequences only^34^. In Table 1, we list the results obtained for all the receiver domains considered in our experimental study. They are sorted from smallest to largest interaction score, and we provide their rank among all the receiver domains involved in *P. brassicacearum* hybrid HKs, as well as the interaction score values, and the score gap with the next ranked one. We also indicated the standard deviation on the interaction scores, and the reliability of our ranking, both obtained by bootstrapping. We found that the top predicted partner for the GacS HK domain is the GacS receiver domain, with a gap substantially larger than the standard deviation, and a reliability equal to 1 (which is the maximum possible). The next predicted potential partners of the GacS HK domain are the receiver domains from 4122, 3082 and 1633. Gaps between their interaction scores are smaller than standard deviations, and accordingly, ranking reliabilities are smaller than 1. In contrast, the gap between the last of these three domains (1633) and the next ranked one (625_2, second receiver of RetS) is substantially larger than standard deviations. In summary, this analysis predicts that the three top potential cross-talk partners for the GacS HK domain among all the receiver domains involved in *P.* *brassicacearum* hybrid HKs, are the receiver domains of 4122, 3082 and 1633. While this approach cannot order them reliably, it predicts that they are consistently better candidates than all other ones. These results are consistent with the phylogeny-based analysis.

**Table 1:** Direct coupling analysis.

Receiver domain	Interaction score	Standard deviation	Gap	Reliability
**4261**	− 275.5	5	22.9	1
**4122**	− 252.6	5.2	4.2	0.7
**3082_3**	− 248.4	5.4	1.5	0.4
**1633**	− 246.9	5.1	18.7	0.4
**625_2**	− 228.2	5.4	0.2	0.2
2955	− 228	5	0.8	0.1
3905	− 227.2	4.8	12.2	0.1
1637	− 215	4.9	1.2	0
**4938**	− 213.8	5.1	0.1	0
**3692**	− 213.7	5	9.1	0
5306	− 204.6	5	0.6	0
**1408**	− 204	5	0.7	0
2831	− 203.3	4.6	3.6	0
3973	− 199.8	4.4	4.7	0
1484	− 195.1	4.9	2.4	0
900	− 192.7	5.3	0.4	0
625_1	− 192.3	5.3	0.1	0

The results show the interaction scores (effective interaction energies) with the kinase domain of GacS. All the receiver domains involved in *P.* *brassicacearum* hybrid HKs were considered, and ranked from smallest to largest interaction scores. The gap corresponds to the difference in interaction score between two successive ranks. The reliability score represents the fraction of bootstrapping replicates where the ranking down to this domain remained identical to the one obtained without bootstrapping (see “Materials and Methods”). The receiver domains belonging to the GacS cluster are marked in bold.

Sequence analysis of the receiver and kinase domains shows amino acid residues common to all hybrid HKs of the GacS cluster, including phosphorylation sites: aspartic acid and histidine residues. Only one residue (asparagine) is common and exclusive to the group of receivers consisting of GacS and the three phylogenetically closest HKs (Supplementary Fig. S2). Coevolution analysis has shown that hybrid HKs do not exhibit the same extended amino acid coevolution between kinase and receiver domains as canonical kinase–receiver pairs^6^.

Collectively, these results suggest the seven hybrid HKs of the GacS kinase cluster share a common evolutionary history (recently duplicated from a common ancestor), and are therefore potentially able to engage in cross-talk/cross-communication with each other. To explore potential phosphotransfer interactions between the different hybrid HKs of the GacS kinase cluster, we focused experimentally on the receiver domains of the hybrid HKs, creating chimeric proteins by replacing the GacS native receiver domain with receiver domains from each of the GacS receiver cluster hybrid HKs.

### Phylogenetic relationships mirror the potential for intramolecular phosphotransfer in hybrid HKs

Nine chimeric GacS hybrid kinases were constructed, seven of which had the GacS receiver domain swapped with other receiver domains from the GacS receiver cluster. The other two chimeras were engineered to contain receiver domains from outside the GacS receiver cluster—the receiver of 2650 (a hybrid HK from outside the GacS cluster) and the first receiver domain of RetS (625_1), only the second receiver domain of which (625_2) belongs to the GacS receiver cluster (Fig. 1). In all chimeras the C-terminal GacS HPt domain was retained, potentially allowing phosphotransfer to GacA when the transplanted receiver domain was able to substitute functionally for the original GacS receiver domain. Expressing the chimeras in a *gacS* mutant and monitoring GacA-dependent phenotypes (protease activity and colony size) thus provided a read-out of whether the chimeric HK was functional.

Table 2 shows the phenotypes of wild-type *P. brassicacearum*, the *gacS* mutant and the *gacS* mutant strain complemented with *gacS*. The wild-type and complemented *gacS* strains showed protease activity and small non-fluorescent colonies, while the *gacS* mutant exhibited no protease activity and large fluorescent colonies due to enhanced pyoverdine production. Three chimeras (containing receiver domains from the three hybrid HKs closest to GacS in the receiver cluster—1633, 3082_3 and 4122) successfully restored wild-type phenotypes, with the exception of the colony size for chimera 1633. In contrast, chimeras harboring receiver domains from RetS-625_2, 1408, 3692 and LadS-4938 displayed *gacS* mutant phenotypes (Table 2). The fact that only three out of seven chimeras whose receiver is phylogenetically close to that of GacS lead to restoration of wild-type strain phenotypes, suggests that the observed phenotypes are not only the result of the high local concentration but also specificity, because four chimeras were ineffective despite their local concentrations likely being high (although we cannot exclude the possibility of misfolding or a high level of dephosphorylation for non-functional chimeras). Taken together, these results suggest that the most phylogenetically similar domains retain the capacity for intramolecular phosphotransfer, a principle we call ‘phylogenetic promiscuity’.

**Table 2:** Phenotypes obtained by expression of GacS chimeras in a *gacS* mutant.

Strains	Protease activity	Colony size
Wild-type	+	Small
*gacS*	−	Large
*gacS*—gacS^+^	+	Small
*gacS*—625_1^+^	−	Large
*gacS*—625_2^+^	−	Large
*gacS*—1408^+^	−	Large
*gacS*—1633^+^	+	Large
*gacS*—2650^+^	−	Large
*gacS*—3082_3^+^	+	Small
*gacS*—3692^+^	−	Large
*gacS*—4122^+^	+	Small
*gacS*—4938^+^	−	Large

### GacS chimeric proteins are capable of both intra- and intermolecular signal transduction

**Figure 3:**
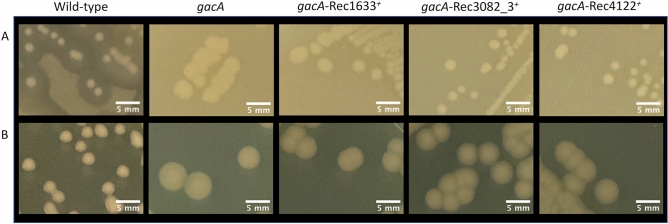
GacA is required for GacS chimeras to restore wild-type phenotypes. Lack of restoration of the wild-type strain phenotypes by expression of GacS chimeras in the *gacA* mutant. Protease activity (lysis zone) on skim milk medium (**A**) and colony appearance on PAF medium (**B**).

As an unorthodox hybrid HK, phosphorylated GacS transfers phosphoryl group intramolecularly to its C-terminal HPt domain, and then intermolecularly to its cognate response regulator GacA. To determine whether this still happens for chimeras that completely (3082, 4122) or partially (1633) restored wild-type phenotypes to the *gacS* mutant (Table 2), GacA-dependence was tested by expressing each of the three chimeras in a *gacA* mutant. This resulted in phenotypes comparable to the control *gacA* mutant (Fig. 3), demonstrating that phosphotransfer to GacA is required for the 1633, 3082 and 4122 chimeras to restore wild-type phenotypes to a *gacS* mutant.

**Figure 4:**
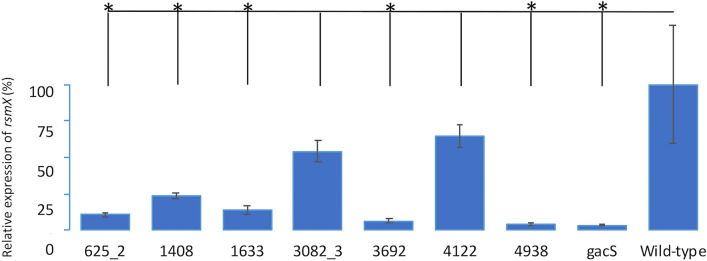
*rsmX* expression levels. Relative expression of *rsmX* for the wild-type strain, *gacS* mutant and *gacS* mutant complemented with one of seven chimeras belonging to the GacS cluster. The reported values represent the means of three experiments, with standard errors indicated by error bars. All data are presented as a percentage relative to the wild-type. Welch’s heteroscedastic F test was performed and * indicates significantly different comparisons (*p* < 0.05) to the wild-type.

As the expression of *rsmX* is completely controlled by the GacS-GacA system^23^, we examined its expression, by quantitative reverse transcription-PCR (qRT-PCR). The *rsmX* expression of the mutant *gacS* harbouring each of the seven chimeras belonging to the GacS cluster was compared to that of the wild-type and the *gacS* mutant. The expression of *rsmX* is significantly higher in the wild-type and 3082–4122 chimeras, than in the *gacS* mutant strain and the four remaining chimeras including 1633, which only partially restored the wild-type phenotypes (Fig. 4, Supplementary Fig. S3).

**Figure 5:**
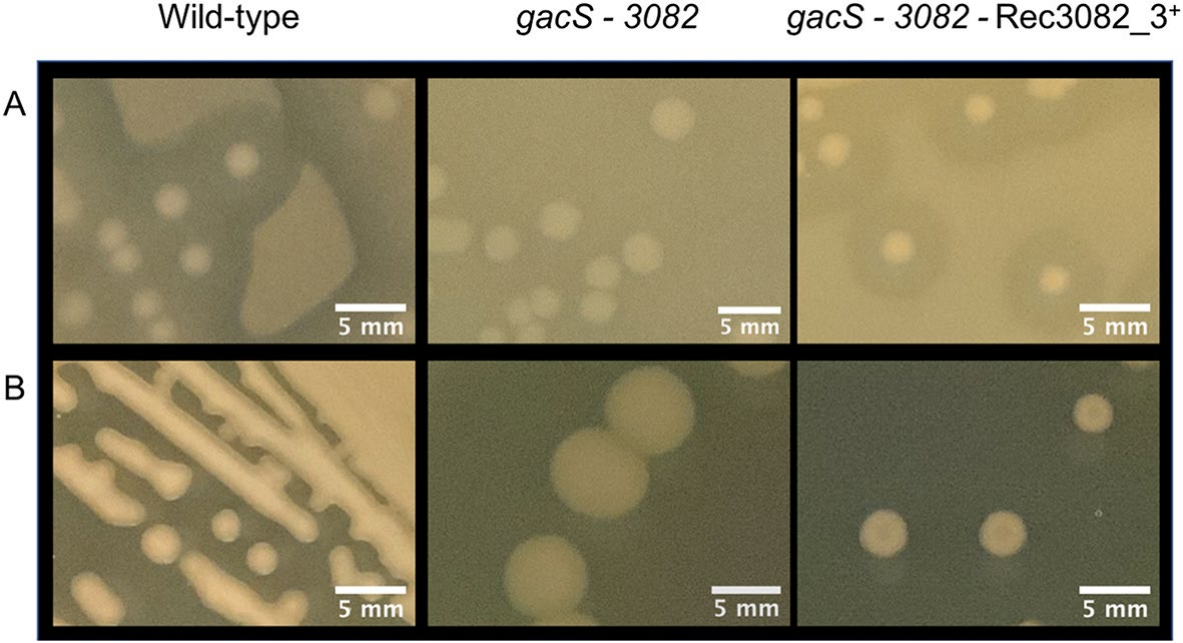
GacS chimeras perform both intra- and intermolecular phosphotransfer. Restoration of the wild-type strain phenotypes by expression of 3082_3^+^ chimera in the *gacS*—*3082* double mutant, compared to the *gacS*—*3082* double mutant. Protease activity on skim milk medium (**A**) and colony appearance on PAF medium (**B**).

Potentially, GacS chimeric proteins might not be fully functional, interacting with, and receiving phosphoryl group from the hybrid HK that had donated the receiver domain to the chimera. This possibility was tested for 3082 by introducing the GacS-3082_3 chimera into the *gacS*—*3082* double mutant, which restored wild-type phenotypes (Fig. 5).

These observations suggest that functional GacS chimeras are able to perform both the intramolecular phosphotransfer reactions (from kinase to receiver to HPt domains) and also the intermolecular phosphotransfer to GacA, as performed by wild-type GacS.

### Transplanted receiver domains in GacS chimeric proteins can be phosphorylated by donor hybrid HKs

**Figure 6:**
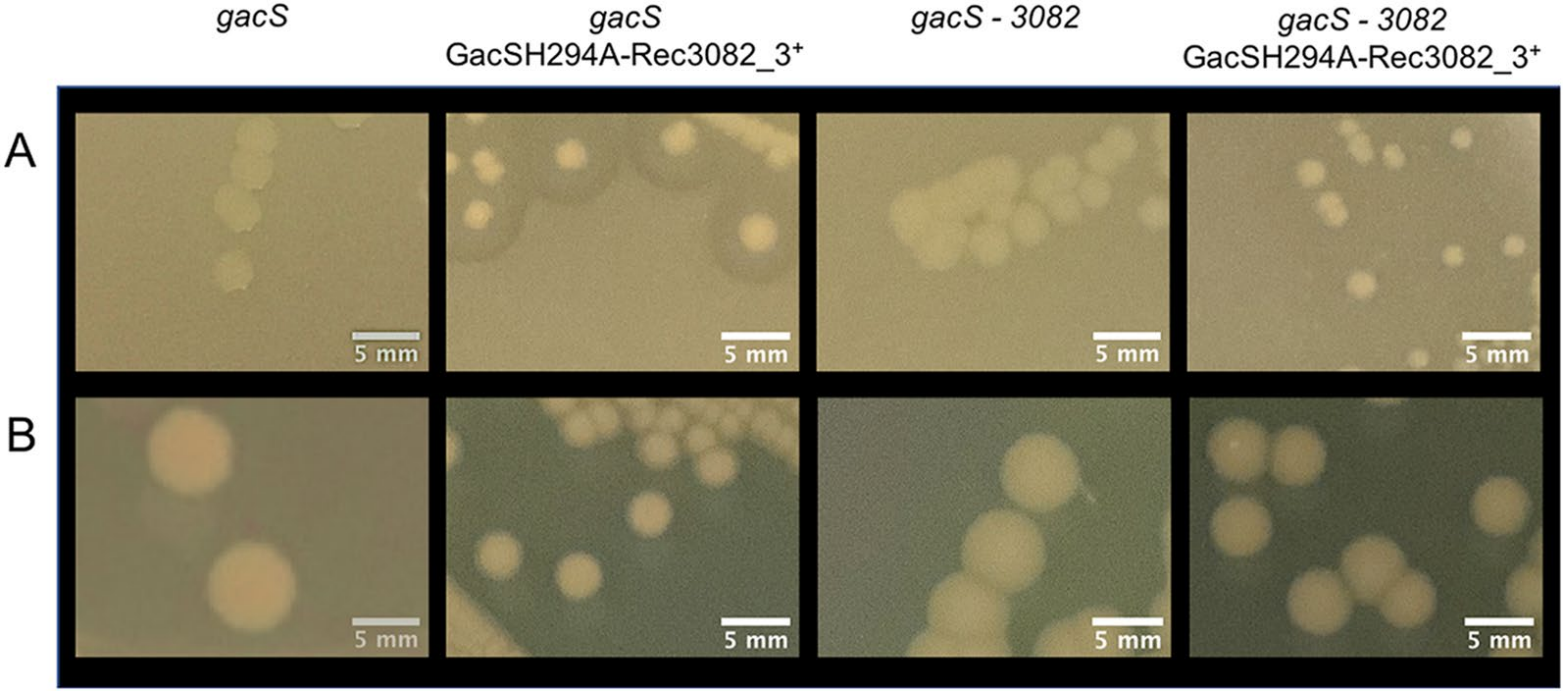
Transplanted receiver domain in a GacS chimera also occurs via cross-talk. Restoration of the wild-type strain phenotypes by expression of GacSH294A—3082_3^+^ chimera in the *gacS* mutant and lack of restoration in the *gacS*—*3082* double mutant. Protease activity on skim milk medium (**A**) and colony appearance on PAF medium (**B**). The phenotypes of the *gacS* mutant and the *gacS*—*3082* double mutant are also shown.

Additionally, it is possible that phosphorylation of the transplanted receiver domain in a GacS chimera also occurs via cross-talk from another TCS (as indeed the native GacS receiver domain can). To test this possibility, we generated a ‘kinase-dead’ GacS-3082 chimera in which the phosphoaccepting histidine residue (H294) in the kinase domain was substituted by alanine, preventing autophosphorylation. Expression of this construct in a *gacS* mutant resulted in restoration of wild-type phenotypes (Fig. 6), suggesting cross-talk between the GacS chimera and another TCS protein. Indeed, expression of the GacSH294A-3082 chimera in the *gacS*—*3082* double mutant did not restore wild-type phenotypes (Fig. 6), implying that it is 3082, which transfers phosphoryl group to GacS-3082 chimeras.

**Figure 7:**
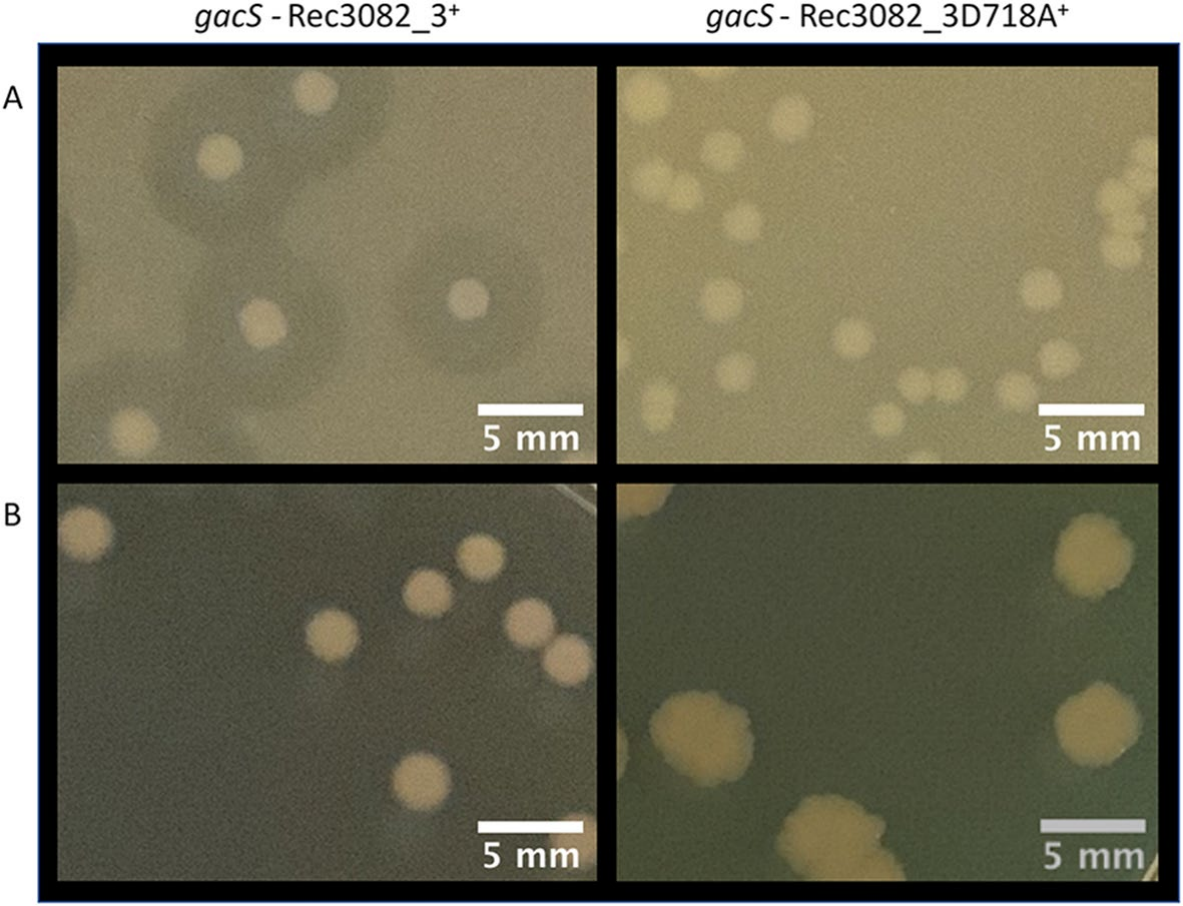
Cross-talk involves the aspartate residue of the transplanted receiver domain. Restoration of the wild-type strain phenotypes by expression of 3082_3^+^ chimera and lack of restoration by expression of 3082_3D718A^+^ chimera in the *gacS* mutant. Protease activity on skim milk medium (**A**) and colony appearance on PAF medium (**B**).

To test whether such cross-talk involves the aspartate residue of the transplanted receiver domain and/or the histidine residue of the GacS HPt domain, a mutant version of the GacS-3082 was engineered in which the aspartate residue in position 718 was substituted with an alanine residue. The GacS-3082D718A construct did not restore wild-type phenotypes to the *gacS* mutant (Fig. 7), likely indicating that the GacS-3082_3 chimera receives phosphoryl group onto the transplanted receiver domain from the 3082 HK.

These observations raised the question of whether the 3082 kinase is involved in the GacS–GacA system, under physiological conditions. To examine this possibility, we constructed a *3082* mutant, which displayed the same phenotypes as the wild-type. This is in accordance with the work of Lee et al.^35^ on *Pseudomonas alkylphenolica*, which showed that the expression of *rsmZ*, one of the final targets of the GacS-GacA system, was not significantly altered in a *bmsA* (orthologous to the *3082* gene) mutant, and overexpression of *bmsA* in a *gacA* mutant did not produce the wild-type phenotype. This does not exclude the possibility that the *3082* gene, and by extension *1633* and *4122* genes, participates indirectly to the GacS-GacA TCS. Indeed, the genomic context of the *3082* gene, for instance, includes three genes encoding CheR (3081), CheB (3080) and a hybrid histidine kinase (3079). This cluster, as well the GacS-GacA system, have been shown to be involved in common processes such as biofilm formation and motility within the *Pseudomonas* genus^35–37^, probably through common mechanisms that have yet to be elucidated.

## Discussion

### Phylogenetic promiscuity

Evolutionary analysis suggested that hybrid HKs were not derived from a common ancestor, and their origin and expansion were achieved by lateral recruitment of a receiver domain into an HK molecule and then duplication as one unit^20^. For example, a comparative study of six species of *Xanthomonas* have shown that the individual domains of a hybrid histidine kinase in one species, originated directly from a gene fusion event that combined cognate HK and RR from closely related species. Such event likely occurs through a mutation of the stop codon, resulting in read-through of the open reading frame^38^. In comparison to orthodox HKs, hybrid HKs have special evolutionary characters, manifested by a higher level of DNA polymorphism and faster evolutionary rate, and frequent gene fusion or fission and duplication^38^.

Consequently, the expansion of hybrid HKs by gene duplication raises the question of their potential cross-talk. Although available data indicate that cross-talk between TCSs generally decreases TCS signaling activity, it cannot be ruled out that such systems could tolerate the presence of more than one interaction partner^39^.

It has been postulated that phylogenetic proximity and the sequence similarity of hybrid HKs are elements that may indicate the degree to which a set of HKs cross-talk or not^10^. The squid symbiont *Vibrio fischeri* uses a signaling network composed of two interacting hybrid HKs to promote biofilm formation and colonization^17^. The receiver domain of hybrid HK RscS and the HPt domain of hybrid HK SypF, interact to phosphorylate two downstream RRs^17^. A second example shows intra- and interprotein phosphotransfer between two hybrid HKs to control the developmental program in *Myxococcus xanthus*^14^. Other examples of interacting hybrid HKs have been described in *Pseudomonas* spp., including the RcsC/PvrS^16^, and GacS multikinase networks. Through GacA activity, the GacS network controls the transcription of several sRNAs, which orchestrate diverse functions in gammaproteobacteria^24,25^. In all these cases, phylogenetic trees of hybrid HK kinase and receiver domains in the P2CS database show that interacting hybrid HKs fall within the same phylogenetic clusters (Supplementary Fig. S4).

In *P. brassicacearum*, similar phylogenetic clustering of hybrid HK kinase and receiver domains is observed, suggesting that they may interact with each other in a multikinase network.

### Interconnection of hybrid HKs

Current knowledge of hybrid HKs, stipulates that a spatial tethering of kinases to their substrates relaxes evolutionary constraints on specificity^6–8^, implying that a covalently linked receiver domain can be functional even in the absence of specific interactions. Nevertheless, using chimeras transplanting the receiver domain of GacS with receiver domains from donor hybrid HKs, we showed that phylogenetic proximity reflects whether a transplant retains biological activity, suggesting interactions were specific (although it is possible non-functional chimeras were misfolded or had high dephosphorylation rates). Interestingly, identification of transmitter–receiver partnerships was more successful for hybrid HKs if domains from hybrid HKs were used as the training set, than if domains from prototypical TCS were used (see “Materials and Methods”). This also suggests that intramolecular interactions within hybrid HKs require specificity, not just tethering of domains to one another.

In addition, the expression of chimeras (with and without mutated kinase domains), in single and double mutant backgrounds, demonstrated that the 3082 third receiver domain can receive signal from both the GacS and 3082 kinase domains, *i.e*. both intra- and intermolecularly. These observations suggest that 1633, 3082 and 4122 are likely targets to be phosphorylated by GacS in vivo*.*

The experiments described in this work investigated flow of signal through the GacS-GacA TCS with domains transplanted into GacS from other TCS proteins. The use of chimeras in mutant backgrounds precluded us from directly defining which/whether specific phosphotransfer interactions occur within/between wild-type proteins. However, in our experiments the signaling domains under study were maintained in the same protein context in which they are found in the wild-type proteins, and the use of chimeras allowed assessment of physiological phenotypes, which bypassed many of the problems associated with in vitro studies involving the phosphorylation of purified proteins^40^.

In *P. aeruginosa*, the hybrid HK RetS attenuates GacS signaling via three mechanisms, including phosphotransfer from the GacS kinase to the second receiver (Rec2) domain of RetS^19^. However, in *P. brassicacearum*, a GacS chimera carrying the Rec2 of RetS (625_2), does not restore wild-type phenotypes to a *gacS* mutant (Table 2). This can be explained by the difference in phylogenetic distance between the two sets of receiver domains. While in *P. brassicacearum* the receivers of the two proteins are in two distinct clusters (Fig. 1), the Rec2 domain of RetS in *P. aeruginosa* is the closest receiver to that of GacS (Supplementary Fig. S4B).

The avoidance of unwanted cross-talk is a major selective pressure for classical HK-RR two-component pathways, driving the diversification of specificity residues to produce post-duplication insulated pathways^41^. However, duplicated hybrid HKs such as those exemplified by the GacS multikinase network, converge towards a common signal output and biological response functions. This convergence allows horizontal interconnection of hybrid HKs, presumably producing sophisticated decision-making networks able to assimilate multiple sensory inputs into a single response^42^. It is also possible that additionally non-hybrid HKs may influence signaling through the network.

It should be noted that the GacS network comprises unusual hybrid sensor kinases that harbor more than one receiver domain. RetS contains two receiver domains, while 3082 contains three. In each hybrid HK, only one of the receiver domains phylogenetically clusters with GacS, or can function within GacS chimeras, implying separate roles and interaction partners for the different receiver domains, as seen for hybrid HKs in similar and diverse organisms^8,19,43^. It is likely that these proteins have multifunctional roles, potentially monitoring multiple environmental stimuli.

### Multikinase network

Through in silico prediction and the use of chimeras, we have identified three proteins (1633, 3082 and 4122) whose receiver domains function interchangeably with that of GacS, enabling signal transduction via GacA and resulting in transcriptional activation of *rsmX*. A receiver domain of one of these proteins, 3082_3, is shown to be able to receive signal (phosphoryl group) from its cognate kinase domain both intra- and intermolecularly, suggesting that 1633, 3082 and 4122 might all be involved in the GacS network in *P.* *brassicacearum*. Further experiments are needed to confirm whether this is the case in vivo, for example by identifying signals which stimulate 1633, 3082 and 4122 signaling and testing for a GacS response to those signals. Our ‘phylogenetic promiscuity’ approach can be used to suggest additional hybrid HKs that may be involved in multikinase networks from publicly available data in the P2CS database. For instance, the *P. aeruginosa* proteins PA_2824 and PA_3462 are likely to be involved in the GacS network^44^, the VF_A0072 protein of *V. fischeri* is likely to interact with the RscS-SypF network^17^, and we would also predict that MXAN_2386 and MXAN_0314 of *M. xanthus* are part of the EspB/EspC network^14^.

The use of evolutionary relationships to predict signaling interactions also implies that signaling relationships can act as indicators of evolutionary heritage. For instance, our results would support a model of GacS network evolution in *P. brassicacearum* by duplication of an ancestral GacS (producing the ancestors of 4122 and 1633) and recruitment of a copy of the GacS receiver domain by another hybrid HK (the ancestor of 3082).

In summary, our results show that the phylogenetic relationships between kinase and receiver domains can be used to guide the rational ‘rewiring’ of communications between and within the HKs forming multikinase networks. This opens possibilities for engineering sophisticated signaling networks by simple domain transplantation, as well as investigating novel functional associations.

## Materials and methods

### Bacterial strains, plasmids and growth conditions

The bacterial strains and plasmids used in this study are listed in Supplementary Table S1. *P.* *brassicacearum* NFM421 and its mutants were grown at 30 °C in tenfold-diluted tryptic soy broth (TSB 1/10) or in 20-fold-diluted tryptic soy broth (TSB 1/20). For detection of extracellular protease activity, bacteria were plated on TSB 1/10 agar plates containing 1% skimmed milk. *Pseudomonas* agar F (PAF) (Difco) was used to reveal colony morphology. *Escherichia coli* strains GM2163, TOP10, and DH5α were grown in lysogeny broth (LB) at 37 °C. For growth on plates, media were solidified with 15 g/l agar (Sigma).

### Construction of mutants

Mutant of *gacA* was generated by deletion mutagenesis as previously described^23^ and *gacS* mutant was developed according to the same procedure, using primers listed in Supplementary Table S2. The mutants obtained by deletion were tested for their phenotypes and then complemented, confirming the expected phenotypes. To construct the *3082* mutant and the *gacS*—*3082* double mutant, the plasmid pCM184^45^, unable to replicate in strain NFM421, was used. To generate a vector carrying only kanamycin resistance, pCM184-Km^r^ was created by eliminating ampicillin and tetracycline resistances from pCM184 through *Sca*I/*Xmn*I and *Eco*RV/*Psh*A1 digestion and re-ligation of the blunt cuts. The plasmid pCM184-Km^r^ was then engineered by inserting an internal fragment of the target gene. Integration of the resulting plasmid into the chromosomal copy disrupts the target gene leading to the production of truncated non-functional proteins. A 0.78 kb fragment, between positions 1190 and 1977 of *3082* gene (Supplementary Table S1), surrounding the kinase domain and including the upstream region of the HATPase domain, was PCR amplified from genomic DNA with the Platinum *Pfx* Polymerase (Thermo Fisher Scientific), using specific primers (Supplementary Table S2) and cloned into the pCR-XL-Topo vector (Thermo Fisher Scientific). After sequence checking, the internal fragment was digested with *Eco*RI, ligated into the *Eco*RI site of pCM184-Km^r^, and used to transform *E*. *coli* strain GM2163 that does not methylate plasmid DNA. The *gacS*—*3082* double mutant is then obtained by triparental conjugation (GM2163 with pCM184-Km^r^ plasmid as a donor, *E. coli* strain DH5α pRK2013 as a helper and the wild-type or the *gacS* mutant as a recipient strain), and screened on TSB 1/10 agar plates supplemented with 100 µg/ml ampicillin (*P.* *brassicacearum* is naturally ampicillin resistant) and 50 µg/ml kanamycin. Mutation of the *3082* gene was confirmed by PCR amplification between the plasmid Km^r^ gene and the chromosomal regions flanking the *3082* gene, then sequencing the PCR amplification products.

### GacS chimeras synthesis and expression

All chimeras consisted of full-length hybrid histidine kinases including transmembrane regions. The GeneArt service (Invitrogen) was used for construction of chimeric protein expression vectors. Briefly, synthetic gene chimeras were assembled from synthetic oligonucleotides and/or PCR products. Synthetic genes were inserted into pMA-RQ (*ampR*), and verified by sequencing before inserting the chimeric genes into the expression vector pME6032^46^. Plasmids were transformed into *E. coli* GM2163 to avoid DNA methylation and then introduced into recipient *P. brassicacearum* strains by tri-partite conjugation (GM2163 with chimeric protein expression plasmids, helper DH5α pRK2013, and the *P. brassicacearum* strain). Potential mutants were obtained by growth on TSB 1/10 agar plates and screening for tetracycline (and kanamycin for the *gacS*—*3082* double mutant) resistant cells.

### Sequence analysis

The software MAFFT (https://mafft.cbrc.jp/alignment/software/) was used to align kinase and receiver domain sequences from HK proteins (http://www.p2cs.org). A threshold of 40% identity across domains was used to identify conserved amino acid residues. The multiple sequence alignment is then visualized using Jalview (https://www.jalview.org).

### qRT-PCR analyses

All the strains that had received the pME6032 plasmids were grown on TSB 1/20 supplemented with isopropyl β-d-1-thiogalactopyranoside (IPTG, 1 mM) and tetracycline (20 µg/ml). Total cellular RNA from 0.5 ml of cultures was extracted using the RNA Protect Bacteria Reagent, the RNeasy Mini Kit and the RNase-Free DNase Set (Qiagen). After a second DNase treatment with the Turbo DNA-free Kit (Ambion), the yield, purity and integrity of RNA were further evaluated using a NanoVue Spectrophotometer (GE Healthcare) and 1% agarose gel electrophoresis. Reverse transcription-PCR (RT-PCR) assays were performed using the Transcriptor First Strand cDNA Synthesis Kit (Roche). Real-time PCR runs were carried out on a LightCycler 480 System (Roche), using the LightCycler 480 Probes Master kit (Roche). The 16S rRNA gene was used as a reference for normalization. Primers and FAM/TAMRA TaqMan probes are listed in Table S2. Fluorescence data were analyzed with the Fit Points method and the efficiencies of qPCR were measured over a dilution range framing our samples (PCR efficiency—*16S rRNA*: 2.012 and *rsmX*: 1.904). To test pairwise differences in *rsmX* expression, we performed Welch’s heteroscedastic F test using the R package ‘onewaytests v2.0’, with a significance threshold to assess statistical difference at *p* < 0.05.

### Phylogenetic analysis of kinase and receiver domains and interaction prediction

The generation of phylogenetic trees was carried out as previously described^10^. Briefly, the kinase and receiver domains of TCS proteins were identified as previously described^47^ and used to construct pairwise alignments using TULIP 1.5^48^, which uses the Smith–Waterman algorithm, with 1000 sequence shuffles, to estimate pairwise Z-values and infer a distance matrix. In the R environment, a hierarchical cluster analysis was undertaken using this distance matrix and the hclust function with ‘Ward’ as a linkage method. An in-house R function then plotted dendrograms to display the hierarchical relationship between domains.

To predict potential interaction partners among paralogs of hybrid HKs in *P. brassicacearum*, we built a direct coupling analysis (DCA) model for hybrid HK kinase and receiver domains using intra-protein pairs between such domains from the P2CS database. Unorthodox proteins were included, but the set of pairs used to construct the model was restricted to those proteins that comprise just one kinase domain and one receiver domain, in order to avoid any ambiguous cases. The resulting training set comprised 12,462 domain pairs. The mean-field approximation was employed to compute DCA couplings between amino acid sites^32–34^, and we used the same alignment methods and parameters as in^34^. Note that in principle we could have used a training set that also included prototypical cognate HK-RR pairs, or a training set based only on prototypical cognate HK-RR pairs, as described previously^49^. However, we found that such a model was less good at predicting intra-protein domain pairs from hybrid HKs than the model constructed using hybrid domains only (the former predicts 29% of all true positives, compared to 96% for the latter), which presumably reflects differences in composition and coevolution between prototypical and hybrid TCSs. The couplings obtained from our DCA model were then employed to compute interaction scores (effective interaction energies) between the *P. brassicacearum* GacS kinase domain and all the receiver domains involved in *P. brassicacearum* hybrid HKs, as previously defined^34^. All the hybrid HKs included in our study were predicted with a 'response_reg' receiver domain, except for HK 2650 which carries a REC domain (data available on www.p2cs.org). This precluded its inclusion in the DCA analysis. To assess the statistical significance of the computed scores, we performed 1000 bootstrapping replicates of the predictions by randomly resampling sequences from the training set (with replacement). This allowed us to compute the variance of each interaction score. For each receiver domain ranked by increasing interaction score with the GacS kinase domain, we further calculated the fraction of bootstrapping replicates where the ranking down to this domain remained identical to the one obtained without bootstrapping. This fraction is indicative of the reproducibility of the ranking by interaction scores, and is called “reliability” in our results.

## Supplementary Information

Supplementary Information.

## Data Availability

Almost all data generated or analysed during this study are included in this published article (and its Supplementary Information files). Upon request raw data would be made available.
