# Supplementary material for: Evolutionary history expands the range of signaling interactions in hybrid multikinase networks
Source: Sci Rep. 2021 Jun 3;11:11763. doi: 10.1038/s41598-021-91260-w (PMC8175716; doi:10.1038/s41598-021-91260-w)
Supplement: Supplementary file 1 — Supplementary Information. [file 41598_2021_91260_MOESM1_ESM.docx]

**Supplementary Information**

**Evolutionary history expands the range of signaling interactions in hybrid multikinase networks**

Philippe Ortet^a^, Sylvain Fochesato^a^, Anne-Florence Bitbol^b,c^, David E Whitworth^d^, David Lalaouna^a,e^, Catherine Santaella^a^, Thierry Heulin^a^, Wafa Achouak^a^, Mohamed Barakat^a,#^

*^a^Aix Marseille Univ, CEA, CNRS, BIAM, LEMIRE, Saint Paul-Lez-Durance, France F-13108*

*^b^Sorbonne Université, CNRS, Laboratoire Jean Perrin (UMR8237), Paris, France F-75005*

*^c^Institute of Bioengineering, School of Life Sciences, Ecole Polytechnique Fédérale de Lausanne (EPFL), CH-1015 Lausanne, Switzerland*

*^d^Institute of Biological, Environmental and Rural Sciences, Aberystwyth University, Ceredigion, SY23 3DD, UK*

*^e^Université de Strasbourg, CNRS, ARN UPR 9002, F-67000 Strasbourg, France*

^#^E-mail for correspondence: mohamed.barakat@cea.fr

**Table S1: Strains and plasmids used in this study**

**Table S2: Primers used in mutagenesis and qRT-PCR analysis (5’ to 3’ orientation)**

| **Gene** | **Primer** | **Sequence** |
| --- | --- | --- |
| Mutagenesis |  |  |
| *Psebr_a3082* | F1189 | AGGAAGTGCTGGCCGAAAC |
|  | R1977 | TTCGAAGATGCTTTGCTGCTG |
| *gacS* | gacS1E | CATAGAATTCCCATGTCGATGATGCGGTCCAC |
|  | gacS2 | GAATCGATACTTGTCTCCTGCATCCAGCGTCTG |
|  | gacS3 | GTATCGATTCCAGGGCAAGATCCAGGGAAC |
|  | gacS4B | CATAGGATCCGTTGGGGAAGGTCAACAGCC |
| qRT-PCR |  |  |
| *rRNA 16S* | 16S-F | CGGAATTACTGGGCGTAAAGC |
|  | 16S-R | CAGTGTCAGTATCAGTCCAGG |
|  | TaqMan-rRNA 16S | CTCAACCTGGGAACTGCATTCAAAACTGTC |
| *rsmX* | rsmX-F | GTTCTGCAGTCCACTGAAGCACAGGAAGT |
|  | rsmX-R | GACCATTACGACTCCCTGTC |
|  | TaqMan-rsmX | CAGGATCAGGGACGATCGACCTTGC |

**FIGURE S1**


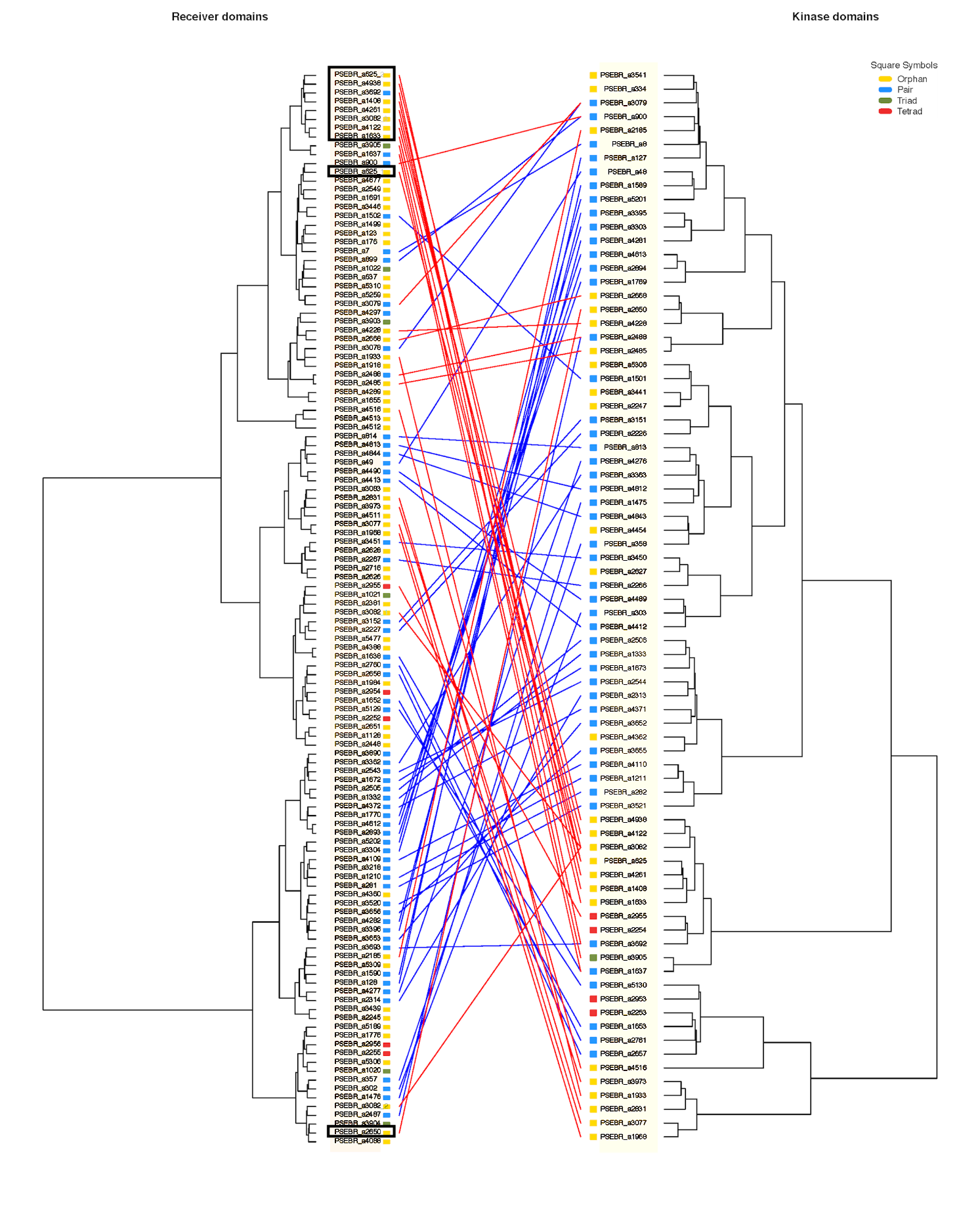


**Figure S1**. **Paired phylogenetic trees from *Pseudomonas brassicacearum***

Blue lines join paired genes. Red lines connect hybrid or unorthodox HKs to their internal receivers. Blue boxes indicate paired genes, while yellow boxes denote orphan genes. Green and red boxes indicate that the gene belongs respectively to a locus containing three (triad) and four (tetrad) TCS genes. Black rectangles highlight the receiver domains of hybrid HKs used in our study. The paired trees are present in the p2cs database as ‘Partnership View’ functionality. The database also contains information on the topology of each protein and displays tools, which allow the user to determine on the fly the topology of each HK.

**Figure S2**

**
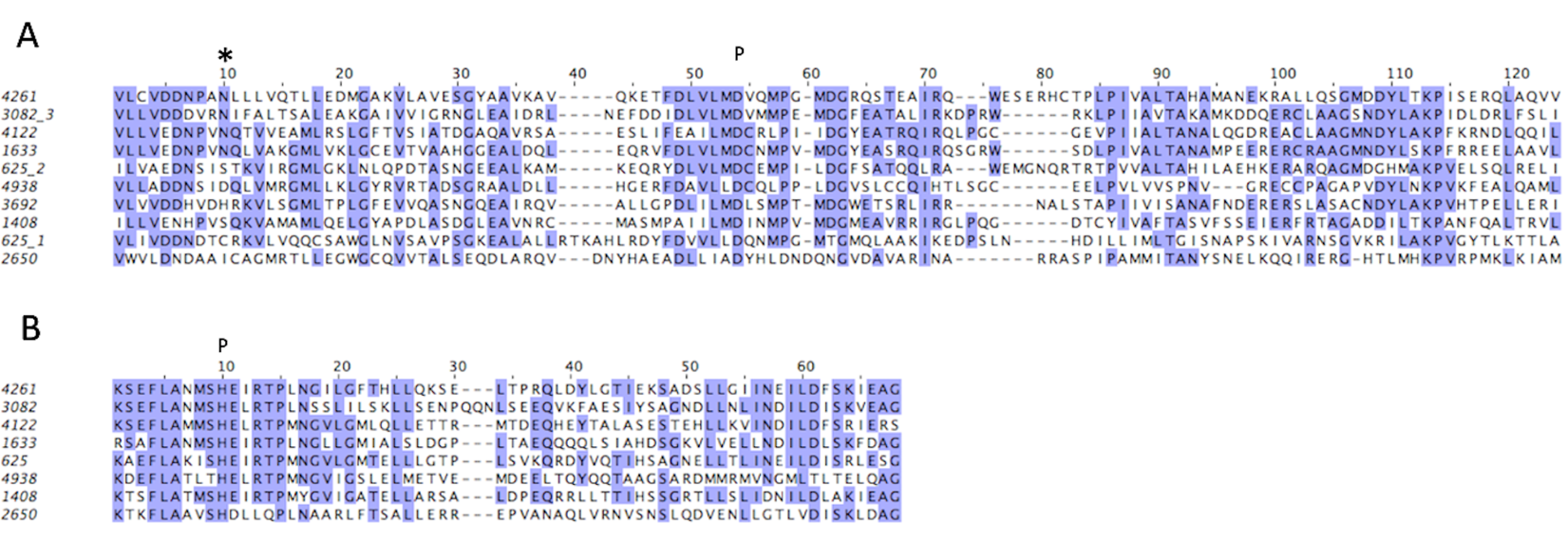
**

**Figure S2 Multiple sequence alignment of the receiver and kinase domains**

The receiver (A) and kinase (B) domains of the HKs belonging to GacS cluster and the two outsiders are aligned using MAFFT. Amino acid residues are numbered according to their position in the domain. Residues highly conserved (at a threshold of 40%) across all receiver and kinase domains are shaded in purple. (*) Conserved residues in GacS and the three phylogenetically closest HKs (3082, 4122 and 1633), (P) phosphorylation sites. The output view is visualized using Jalview.

**Figure S3**


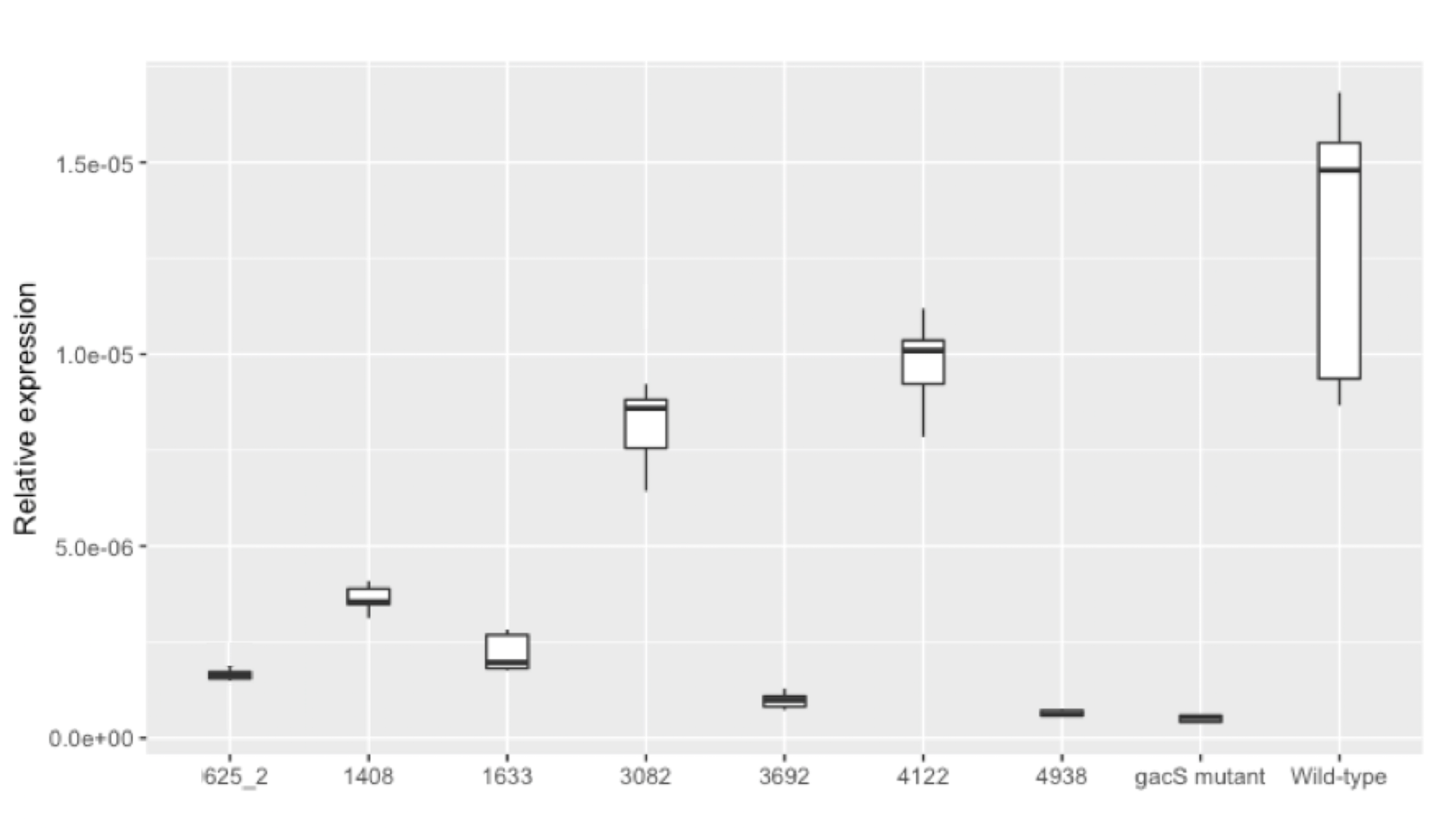


**Figure S3**. **Boxplots of the Welch’s heteroscedastic F test**

The test was performed on the *rsmX* gene expression data, using the R package ‘onewaytests’. The result of the statistical test is reported in Figure 4.

**Figure S4**

**
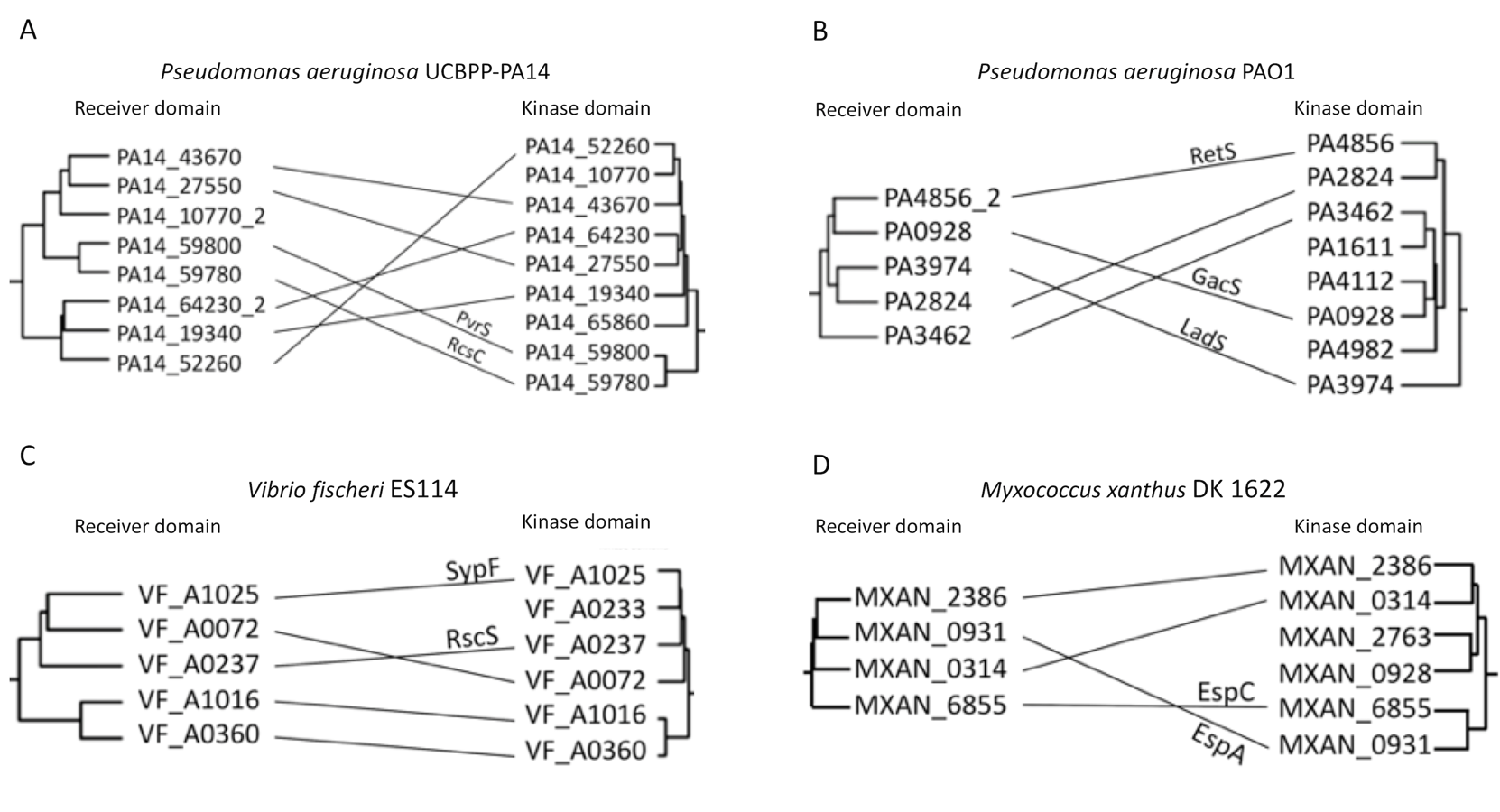
**

**Figure S4**. **Paired phylogenetic trees**

*Pseudomonas aeruginosa* PA14, focus on the ‘PvrS-RcsC cluster’ (A). *Pseudomonas aeruginosa* PAO1, focus on the ‘GacS cluster’ (B). *Vibrio fischeri* ES114, focus on the ‘SypF-RscS cluster’ (C). *Myxococcus xanthus* DK 1622. focus on the ‘EspA-EspC cluster’ (D). Black lines connect hybrid HKs to their internal receivers.
